# Spatiotemporal Dynamics of Assyrtiko Grape Microbiota

**DOI:** 10.3390/microorganisms12030577

**Published:** 2024-03-14

**Authors:** Konstantinos Tegopoulos, Theodora Tsirka, Christos Stekas, Eleni Gerasimidi, George Skavdis, Petros Kolovos, Maria E. Grigoriou

**Affiliations:** Department of Molecular Biology & Genetics, Democritus University of Thrace, 68100 Alexandroupolis, Greece; konstego1@mbg.duth.gr (K.T.); ttsirka@mbg.duth.gr (T.T.); elgerasimidi@gmail.com (E.G.); gskavdis@mbg.duth.gr (G.S.)

**Keywords:** microbiome, bacteria, fungi, *Vitis vinifera*, Assyrtiko, metagenomic study, microbial *terroir*

## Abstract

*Vitis vinifera*, an economically significant grapevine species, is known for wine, juice, and table grape production. The berries of wine grapes host a diverse range of microorganisms influencing both grapevine health and the winemaking process. Indigenous to Greece, the emblematic variety Assyrtiko, renowned for high-quality white wines, originated from Santorini and spread to various Greek regions. Despite existing studies on the microbiota of several varieties, the carposphere microbiota of Assyrtiko grapes remains unexplored. Thus, we conducted a spatiotemporal metagenomic study to identify the epiphytic microbial community composition of Assyrtiko grapes. The study was conducted in two consecutive vintage years (2019 and 2020) across three different and distinct viticulture regions in Greece (Attica, Thessaloniki, Evros). We performed amplicon sequencing, targeting the 16S rRNA gene for bacteria and the ITS region for fungi, with subsequent comprehensive bioinformatic analysis. Our data indicate that the distribution and relative abundance of the epiphytic carposphere microbial communities of the Assyrtiko variety are shaped both by vintage and biogeography.

## 1. Introduction

*Vitis vinifera*, a widely cultivated grapevine species, holds immense economic importance due to its role in the production of wine, juice, and table grapes [1,2]. The quality and distinctive attributes of wine not only stem from the grapevine variety but are profoundly influenced by both the vine *terroir* and microbial *terroir*. The former encompasses the physical and environmental factors of the vineyard, while the latter includes the microbial communities present in the grapevine and the surrounding soil [3]. Microbial *terroir* directly impacts characteristics of the wine, such as the flavor, aroma, and quality [4,5], through a process that initiates in the vineyards and further evolves during fermentation [6,7,8].

The rhizosphere, phyllosphere, and mainly the grape berries are important habitats shaping the microbial *terroir* [9]. These intricate microbial communities encompass a diverse set of yeasts, filamentous fungi, and bacteria [10]. A number of these microorganisms play essential roles promoting plant growth, fruit yield, pathogen defense, crop health and survival [11,12,13] while others may negatively affect the health of the plant by causing serious diseases, which result in economic loss due to a decrease in the yield and/or quality [9,14]. For example, the most known diseases of vine plant and grapes, namely downy mildew, powdery mildew, and grey rot, are caused by the phytopathogenic taxa *Plamospora viticola*, *Erysiphe necator,* and *Botrytis cinerea*, respectively [15]. It is important to highlight that, although these species are ethanol intolerant and thus unable to grow in the wine, they significantly affect its quality through grape damage [11].

Concerning the structure of grape microbial communities, previous studies have identified that *Proteobacteria, Firmicutes*, *Actinobacteria*, *Acidobacteria,* and *Bacteroidetes* are the predominant bacterial phyla, while members of the *Pseudomonas*, *Bacillus*, *Serratia,* and *Sphingomonas* appear to be among the dominant taxa as well [16,17]. In terms of fungal diversity, *Ascomycota* dominate at a phylum level, followed by *Basidiomycota* [17]. Other frequently detected fungal taxa include *Aspergillus*, *Alternaria*, *Aerobasidium*, *Davidiella*, *Erysiphe* [3,6]. Interestingly, the sugar fermenting yeast *Saccharomyces cerevisiae* is rarely found grape berries [11].

Vineyard and wine microbial diversity has been investigated using both culture and non-culture approaches [18]. Cultivation-dependent methods, such as restriction fragment length polymorphism (RFLP) and random amplified polymorphic analysis (RAPD), are characterized as time consuming and having poor reliability [18]. As a result, within the last two decades, DNA fingerprinting methods, such as denaturing gradient gel electrophoresis (DGGE) and terminal restriction fragment length polymorphisms (T-RFLP), have been extensively applied to explore the microbial communities in grapevine samples [19,20,21]. Despite having some advantages in speed and accuracy over culture methods, the aforementioned approaches possess a limited ability to detect certain or low-abundance taxa [21]. These limitations, combined with the development of Next Generation Sequencing (NGS) techniques, have prompted scientists to study microbial communities at a higher resolution using metagenomic approaches [21]. This approach provides a more comprehensive understanding of microbial diversity, including the detection of low-abundance taxa, thereby enabling researchers to study the structure of microbial communities in greater detail and with higher resolution [5,22,23]. High-throughput sequencing techniques have been extensively employed in the analysis of microbial communities in various environmental niches, including grape, must, and wine [13]. However, as this approach is relatively recent, its potential impact in this field remains to be fully explored [21].

Recent studies highlight that the microbial *terroir* of grapevines is influenced by various factors, such as grapevine genotype, geographical location, and agricultural practices [22,23]. When considering grapevine genotype, microbial communities exhibit significant differences among various *Vitis vinifera* varieties, as evidenced by a study involving 36 different varieties cultivated within the same vineyard [24]. Furthermore, there is evidence of geographic correlation in the structure of microbial community across vineyards in different countries, including the USA, China, and Spain [13,23,25]. These findings suggest that microbial communities associated with grapevines are influenced by geographical factors, and highlights the need for region-specific studies to better understand the microbial *terroir* of grapevines.

In Greece, viticulture has a rich history, dating back to ancient times having a vital role in the country’s cultural heritage and economy [26]. Among the most prominent Greek grapevine varieties is the emblematic Assyrtiko, which originates from the island of Santorini, but is also cultivated in other regions of Greece as well as in other countries. Assyrtiko is highly valued for its capacity to produce top-quality white wines with distinctive mineral and citrus aromas, and it is often used in blends with other grape varieties [27]. Despite the economic and cultural significance of Assyrtiko, there is limited information available regarding the microbial *terroir* of its vineyards across Greece. This information could serve as a stepping stone toward understanding the influence that grape microbiomes may have on the organoleptic characteristics of wine, particularly in the context of spontaneous wine fermentations. In recent years, there has been a growing trend among winemakers to produce wines with distinct regional aromas and tastes, making this knowledge increasingly valuable.

In this work, we analyze the structure of the grape microbial community of Assyrtiko variety across diverse regions and timepoints in Greece. To this end we performed amplicon sequencing of the 16S rRNA gene for bacterial microbiome analysis and of the ITS2 region for fungal microbiome followed by advanced bioinformatic analysis. Our sampling spanned across Assyrtiko vineyards in three distinct Greek regions over two vintages. Through comprehensive data analysis, our spatiotemporal analysis identified dominant and unique taxa while revealing region-specific and time-specific patterns of microbial diversity associated with the Assyrtiko vineyard *terroir*, providing useful insights into the factors that contribute to the grapevine’s health and the production of high-quality wines.

## 2. Materials and Methods

### 2.1. Sample Collection

In total, 24 grapevine (var. Assyrtiko) samples were collected at harvest, over two years (2019 and 2020) from three different vineyards in Greece: Attica, Thessaloniki, and Evros. The exact coordinates of the sampling sites, the distances between them as well as their position on the map are presented in Supplementary Table S1 and Supplementary Figure S1 (created using QGIS–v.3.30.1), respectively. At each site, four samples were collected from distal spatial points of different vines just before harvesting under sterile conditions. The samples, representing four biological replicates, were collected and processed independently. Thus, each vineyard contributed eight samples, four from 2019 and four from 2020. To assess the sugar content in the harvested grapes, Baumé degrees of each sample were quantified using a calibrated Baumé hydrometer. The Baumé measurements exhibited a consistent range, ranging from 11 to 13 degrees across all samples.

### 2.2. Next Generation Sequencing

#### 2.2.1. DNA Extraction

To release microorganisms from the surface, 120 g of grapes were placed in a sterile 500 mL flask containing 80 mL NaCl (0.9% *w*/*v*) and washed for 3 h at 120 rpm at room temperature (RT). Then, the grapes were discarded and the washing solution was centrifuged at 5000× *g* for 15 min (4 °C). The supernatant was discarded and the pellets were resuspended in 100 μL of sterile water. Subsequently, the samples were homogenized using the Precellys Evolution Homogenizer (Bertin Instruments, Montigny-le-Bretonneux, France) to facilitate cell lysis. DNA extraction was performed with the NucleoSpin Soil kit (Macherey-Nagel, Düren, Germany) following the supplier’s instructions. DNA concentration and purity were assessed using NanoDrop Spectrophotometer (Thermo Fisher Scientific, Waltham, MA, USA). DNA integrity was assessed using gel electrophoresis in a 0.8% *w*/*v* agarose (UltraPure Agarose, Invitrogen, Carlsbad, CA, USA) gel pre-stained with GelRed Nucleic Acid Gel Stain (Biotium, Fremont, CA, USA).

#### 2.2.2. Amplicon Amplification with PCR

For each sample, two PCR reactions were conducted, targeting: (a) the fungal Internal Transcribed Spacer 1 (ITS1) and (b) the bacterial hypervariable region V3 of the 16S rDNA gene. The ITS1 region was amplified using the primers 5′-ACCTGCGGARGGATC-3′ (F) and 5′-GAGATCCRTTGYTRAAAGTT-3′ (R), while the V3 region was amplified using the primers 5′-ACTGAGACACGGTCCAGACT-3′ (F) and 5′-GTATTACCGCGGCTGCTG-3′ (R). The amplification process was carried out in a VeritiPro Thermal Cycler (Thermo Fisher Scientific, Waltham, MA, USA), where each reaction, with a total volume of 20 μL, comprised 10 μL of KAPA SYBR FAST qPCR Master Mix (2×) (Sigma-Aldrich, St. Louis, MO, USA), 0.1 μΜ of each primer, and 50 ng of genomic DNA as template. For the ITS1 amplification, an initial denaturation step at 95 °C for 5 min was followed by 30 cycles of denaturation at 95 °C for 30 s; annealing at 57 °C for 30 s; extension at 72 °C for 30 s; and a final extension step at 72 °C for 5 min. For the PCR targeting the bacterial V3 region, an initial denaturation step at 95 °C for 5 min was performed; followed by 30 cycles of denaturation at 95 °C for 30 s; annealing at 58 °C for 30 s; extension at 65 °C for 30 s; with a final extension at 65 °C for 5 min. Successful amplification was evaluated through electrophoresis in 2% *w*/*v* agarose gels pre-stained with GelRed Nucleic Acid Gel Stain (Biotium, Fremont, CA, USA).

#### 2.2.3. Library Preparation and Sequencing

For the purification of the PCR products, we employed NucleoMag NGS Clean-up and Size Select magnetic beads (Macherey Nagel, Düren, Germany), adhering to the manufacturer’s recommended procedures and maintaining an 1/1.8 volume ratio (DNA/beads). Following this purification step, we utilized a Qubit 4 Fluorometer (Thermo Fisher Scientific, Waltham, MA, USA) in conjunction with the Qubit dsDNA HS Assay Kit to evaluate the concentration of the purified PCR products.

Following the purification, we proceeded to create barcoded libraries, utilizing 100 ng of the purified PCR products as a template. This process was performed using the Ion Plus Fragment Library Kit (Thermo Fisher Scientific, Waltham, MA, USA) in accordance with the specifications provided by the manufacturer. The DNA of the libraries underwent purification, ensuring a 1/1.4 (DNA/beads) volume ratio, with NucleoMag NGS Clean-up and Size Select magnetic beads (Macherey Nagel, Düren, Germany). Subsequent to this, quantitation of DNA for each library was performed through qPCR, employing the Ion Universal Library Quantitation Kit (Thermo Fisher Scientific, Waltham, MA, USA). Moving forward, 100 pM of purified library DNA was processed in the Ion OneTouch™ 2 System (Thermo Fisher Scientific, Waltham, MA, USA) for template preparation and objected to sequencing with Ion Torrent GeneStudio S5 (Thermo Fisher Scientific, Waltham, MA, USA), as per the provided manufacturer’s instructions.

### 2.3. Data Analysis

The initial processing of raw sequencing data involved the removal of polyclonal, low-signal, and low-quality reads. Subsequently, the obtained UBAM files were converted to fasta format files using samtools (v.1.13) [28]. The analysis of amplicon sequencing data was conducted using Mothur (v.1.45.3) [29] and resulted in the exclusion of reads with ambiguous bases, homopolymers, and improper lengths. The reads were then aligned and classified into Operational Taxonomic Units (OTUs) (Tables S2 and S3) using the bacterial Greengenes (v.13_8_99) and fungal UNITE (v.6) reference databases. Classified reads with at least 97% sequence similarity were clustered into the same OTU. Subsequently, a 0.1% filter with a 20% prevalence rule was applied to filter out low-abundance bacterial and fungal OTUs. Analyses including rarefaction analysis, calculation of the Shannon and Simpson indices, and core microbiome identification were conducted using the Microbiome Analyst online platform [30] (available at: https://www.microbiomeanalyst.ca/, accessed on 10 February 2024). The same platform was employed for creating heatmaps and relative abundance graphs. Finally, to further explore potential differences in the structure of microbial communities, Principal Component Analysis (PCA) based on unweighted Unifrac distance was performed using in-house R scripts and the DESeq2 [31] R library.

## 3. Results

### 3.1. Geographic Distribution

To investigate the microbial community structure, encompassing both fungi and bacteria within the Assyrtiko grapevine variety across Greece, a spatiotemporal study was conducted. The study focused on three geographically diverse regions with a history of active viticulture since ancient times: Attica (southern part), Evros (northeastern part), and Thessaloniki (northern part). Spanning two consecutive years (2019 and 2020), the study collected four samples from each region and vintage.

### 3.2. Diversity Assessment

#### 3.2.1. Bacterial Communities

The analysis of 16S rDNA sequences from 24 grapevine samples (var. Assyrtiko) resulted in 514 operational taxonomic units (OTUs) at a 97% similarity threshold. Using a 0.1% filter with a 20% prevalence rule, the analysis identified 127 bacterial OTUs (Table S2) meeting this criterion, excluding 387 low-abundance OTUs.

**Figure 1:**
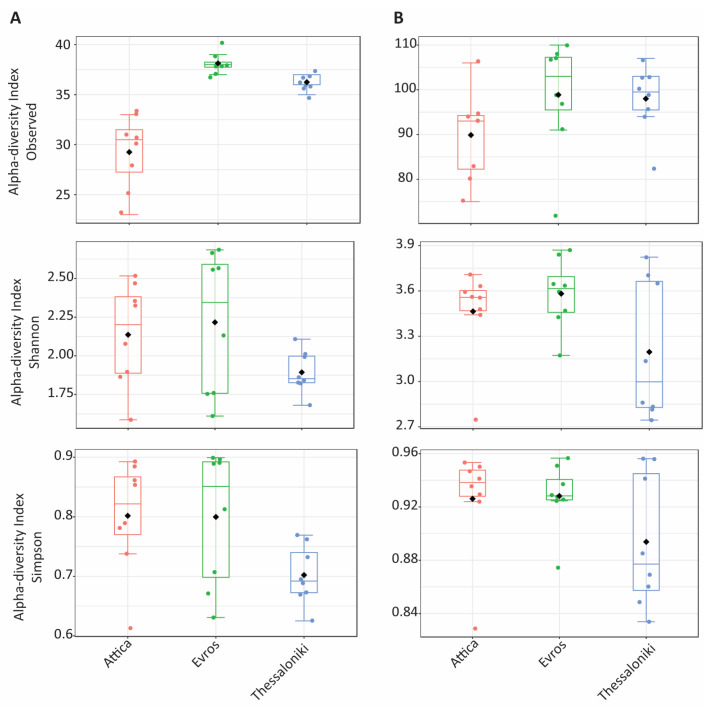
Values of Observed species, Shannon and Simpson indices across fungal (**A**) and bacterial (**B**) communities.

Alpha diversity slightly varied across datasets (Figure 1, Table 1). Samples from Evros 2019 (EV19) exhibited the highest number of observed OTUs, whereas Attica 2019 (AT19) recorded the lowest per-sample OTU count (Table 1). Despite these disparities, when assessing the Shannon and Simpson indices—combining species richness and evenness—no significant differences between samples were noted, except for Thessaloniki in 2019 (TH19) (Table 1).

#### 3.2.2. Fungal Communities

The analysis of ITS2 sequences from the same samples resulted in 374 OTUs at a 97% similarity threshold. Applying a 0.1% filter with a 20% prevalence rule excluded 333 fungal OTUs, leaving 40 that met the specified criteria.

Diversity within fungal communities fluctuated among the studied datasets (Figure 1, Table 2). Interestingly, the highest average of observed OTUs concerned the EV20 samples, in contrast to bacterial communities (Table 1). However, the Shannon and Simpson diversity indices agreed that vintage influenced fungal communities, with samples collected in 2020 exhibiting greater diversity in all regions (Table 2).

### 3.3. Taxonomic Distribution and Comparison

#### 3.3.1. Bacterial Microbiome

**Figure 2:**
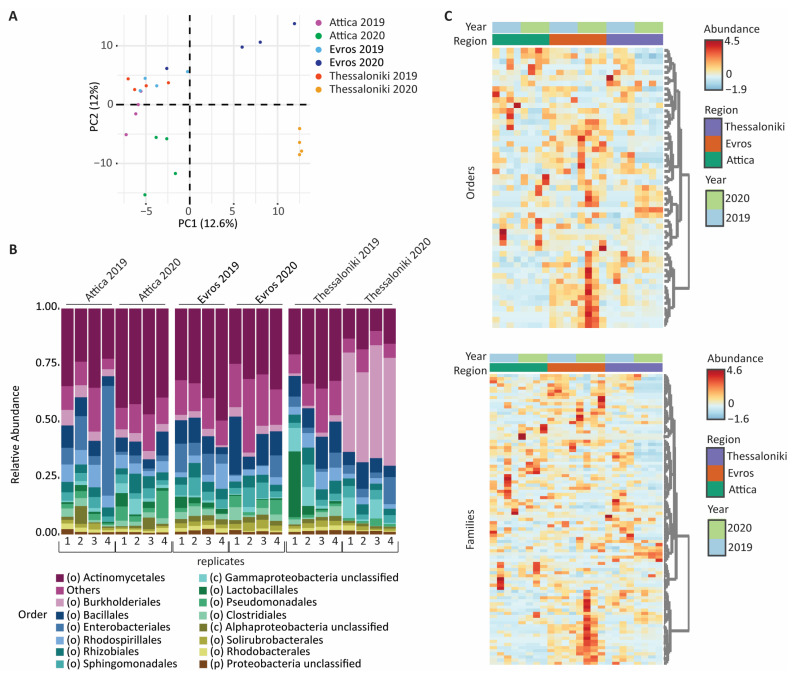
(**A**) Principal Component Analysis (PCA) based on the unweighted Unifrac distance (**B**) Relative abundance plots of bacterial OTUs at order level (**C**) Feature scaled heatmap illustrating the bacterial taxonomic abundance profiles across the studied samples at order (**top**) and family (**bottom**) level.

PCA analysis based on the unweighted Unifrac distance was performed to investigate whether there were any discernible patterns or separation among the samples based on their geographic distribution and/or year of vintage (Figure 2A). Interestingly, the samples from the three regions collected in 2019 clustered relatively close to each other, unlike those collected in 2020, which showed significant differences between different regions. When comparing the pair of samples from the same regions over the two vintages, pronounced differentiation was observed, especially in the Thessaloniki region and, to a lesser extent, in Attica and Evros. Overall, the structure of bacterial communities appeared to be more influenced by vintage and less by the geographic component. Subsequently, we compared the relative abundances of the OTUs at the order level within the bacterial communities (Figure 2B). In the overwhelming majority of samples, the order *Actinomycetales* appeared as the dominant order, with only Thessaloniki 2020 samples showing dominance of the order *Burkholderiales*. However, across all samples, the most abundant orders were predominantly common, suggesting that both vintage and geographical location influence the composition of communities in lower abundance operational taxonomic units.

Furthermore, despite the common dominance of orders (Figure 2B) and the overall similar number of observed OTUs between datasets (Table 1), the distinct patterns observed across the different samples in the heatmaps (Figure 2C) indicate that the differences between the studied datasets involve the presence of low-abundance OTUs. The bacterial microbiome of the grape’s surfaces appeared to be more influenced by the vintage, with changes predominantly affecting non-dominant taxonomic units, except for the samples from Thessaloniki in 2020.

**Figure 3:**
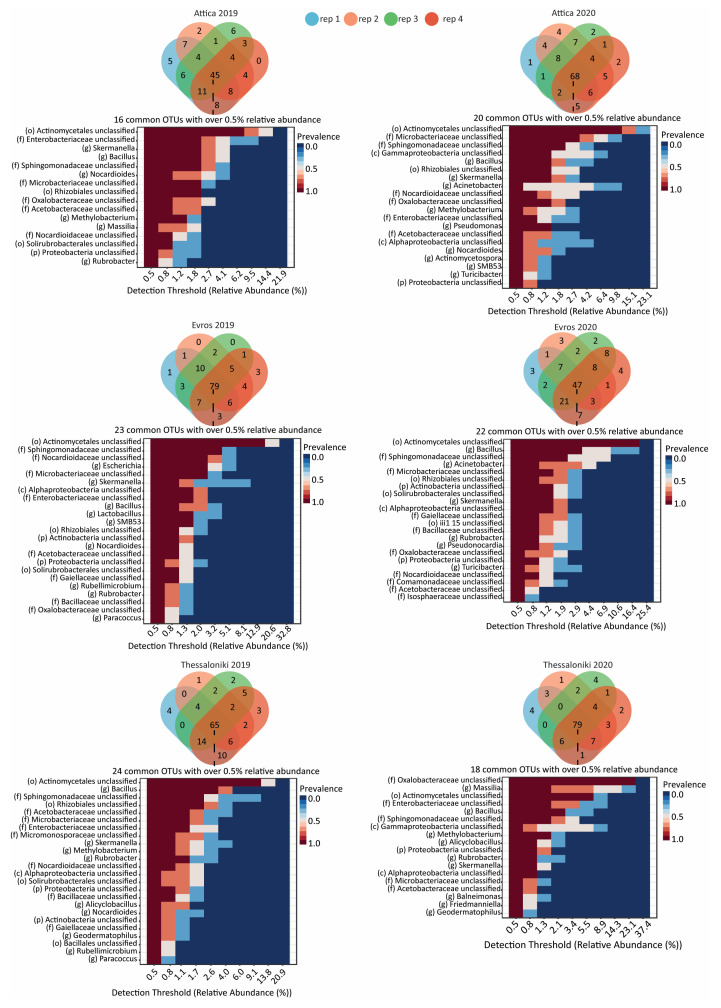
Venn diagrams showing shared OTUs within each vineyard and vintage, based on the analysis of four replicates. Below each diagram, the relative abundance and prevalence of bacterial OTUs exceeding 0.5% across the sampled datasets is shown.

In addition, we identified the core bacterial microbiome for each region and vintage (Figure 3). Interestingly, compared to the total OTUs identified, the core microbiome of each dataset consisted of a considerably smaller number of OTUs (Table 2). In the case of Attica samples, a total of 114 and 119 OTUs were identified for 2019 and 2020, respectively; the core microbiome of 2019 comprised of 45 OTUs (39.4%) while that of 2020 of 68 OTUs (57.1%). For Evros samples, the core microbiomes for 2019 and 2020 consisted of 79 OTUs (63.2%) and 44 OTUs (36.9%), respectively. Notably, Thessaloniki’s core microbiome, with 65 OTUs (54.1%) for 2019 and 73 OTUs (62.4%) for 2020, maintaining a relatively consistent number of OTUs across the two years, despite the significant differences in the abundance of the dominant orders. Notably, only a small fraction of the OTUs of the core microbiome was detected with an abundance higher than 0.5% (16 for AT19, 20 for AT20, 23 for EV19, 22 for EV20, 24 for TH19 and 18 for TH20); this observation may explain the significant differences in the number of taxa comprising the core bacterial microbiome observed between vintages, as lower abundance taxa are more susceptible to environmental changes.

**Figure 4:**
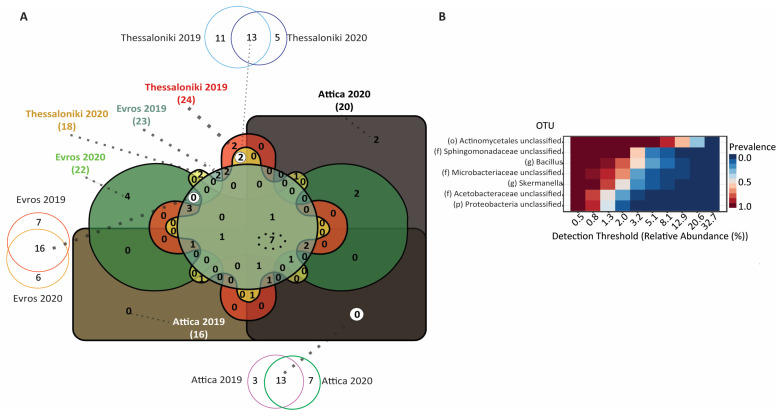
(**A**) Non-symmetric Venn diagram illustrating common and unique bacterial OTUs within the different datasets. The core microbiome for each region in the two vintages, identifying the number of OTUs consistently present in the same vineyard/region over both years, is shown in the three symmetrical Venn diagrams (**top**, **bottom**, **left**). The number of OTUs unique for a region is shown in white circles in the non-symmetrical Venn diagram. (**B**) A diagram showing the relative abundance and prevalence of the core Assyrtiko microbiome (7 OTUs identified on grapes of all regions and vintages).

We then sought to identify the region-specific core microbiome, i.e., OTUs detected irrespective of the vintage (Figure 4). The analysis of each dataset’s core microbiome unveiled that the region-specific core microbiome of Assyrtiko encompasses, as expected, a fraction of the shared taxonomic units over the 2-year vintages. More specifically, 81.5% of the shared OTUs in samples AT19 (13 out of 16) and 65.0% (13 out of 20) in AT20 form the core microbiome of Attica (13 out of 23 OTUs). Regarding Evros, 69.5% of EV19 and 72.7% of EV20 OTUs form the core microbiome of this region (16 out of 29 OTUs), while 54.1% of TH19 and 72.2% of TH20 form the core microbiome of the Thessaloniki region (13 out of 29 OTUs).

Moreover, we explored the presence of OTUs exclusively detected in each region, regardless of the vintage, aiming to unveil a potential region-specific bacterial signature. Interestingly, we identified 2 OTUs unique to the Thessaloniki region (*Geodermatophilus*, *Alicyclobacillus*), while Attica and Evros exhibited none (Figure 4). Finally, we conducted a comparative analysis of the core bacterial microbiomes across all regions and for both timepoints. The results, shown in the non-symmetrical Venn diagram, revealed a set of 7 bacterial OTUs, comprising the core microbiome of the Assyrtiko variety present on the surface of grapes irrespective of geographic region and vintage (Figure 4).

#### 3.3.2. Fungal Microbiome

**Figure 5:**
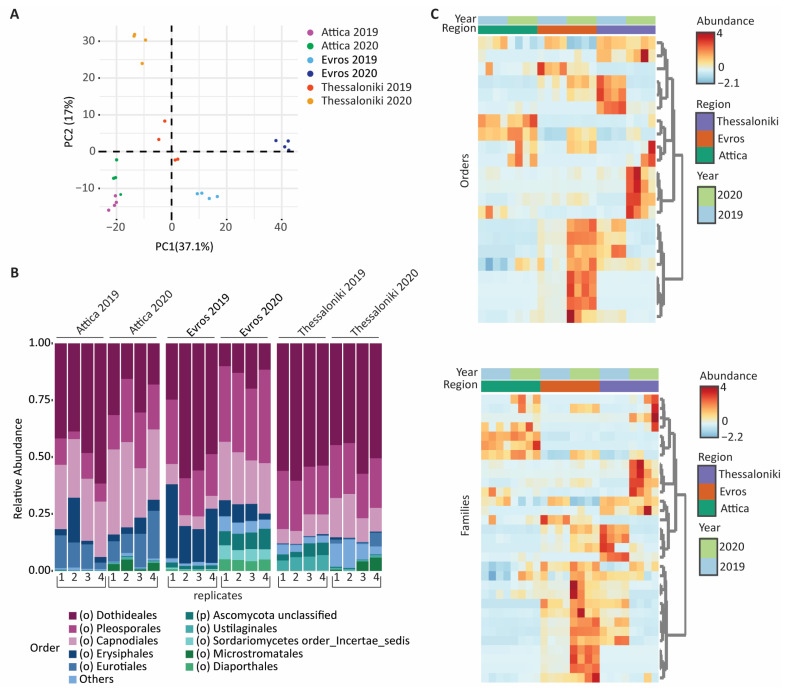
(**A**) Principal Components Analysis (PCA) based on the unweighted Unifrac distance (**B**) Relative abundance plots of fungal OTUs at order level (**C**) Feature scaled heatmap illustrating the fungal taxonomic abundance profiles across the studied samples at order (**top**) and family (**bottom**) level.

We conducted a PCA analysis based on the unweighted Unifrac distance to unveil separation in the samples based on their geographic distribution and/or vintage (Figure 5A). The samples from Attica clustered closely together, whereas in the cases of Thessaloniki and Evros differences between the two vintages were observed. In contrast to the bacterial microbiome, the composition of fungal communities appeared to be influenced by both the geographical location and the vintage.

Subsequently, we compared the relative abundance of the fungal OTUs at the order level (Figure 5B). Except for the order Microstromatales, detected only in AT20, the analysis of the samples from Attica revealed a significant number of common orders, among which we observed differences in their abundance between the two vintages. In the case of samples from Evros region, significant differences were detected in the abundance of common orders, such as *Ustilaginales*, *Sordariomycetes,* and *Diaporthales*, with an increased abundance values in the second vintage (EV20). On the other hand, in the case of Thessaloniki, although we did not detect significant differences in the abundance of the dominant orders, significant differences were observed in low-abundance orders in between vintages (TH19 and TH20). We then compared the abundance of the identified orders and families (Figure 5C). With the exception of *Dothideales*, *Capnosporales,* and *Pleosporales* which appeared as the dominant orders across all samples, the majority of the fungal families identified for a specific region were either not detected or detected in significantly lower abundance in other regions, suggesting that specific clusters of fungal families characterize each region. Furthermore, the OTU clusters indicated that samples from Attica show higher levels of uniformity compared to those of Evros and Thessaloniki, in which we identified distinctive patterns. These findings are supported by the results of the relative abundance diagrams (Figure 5B).

**Figure 6:**
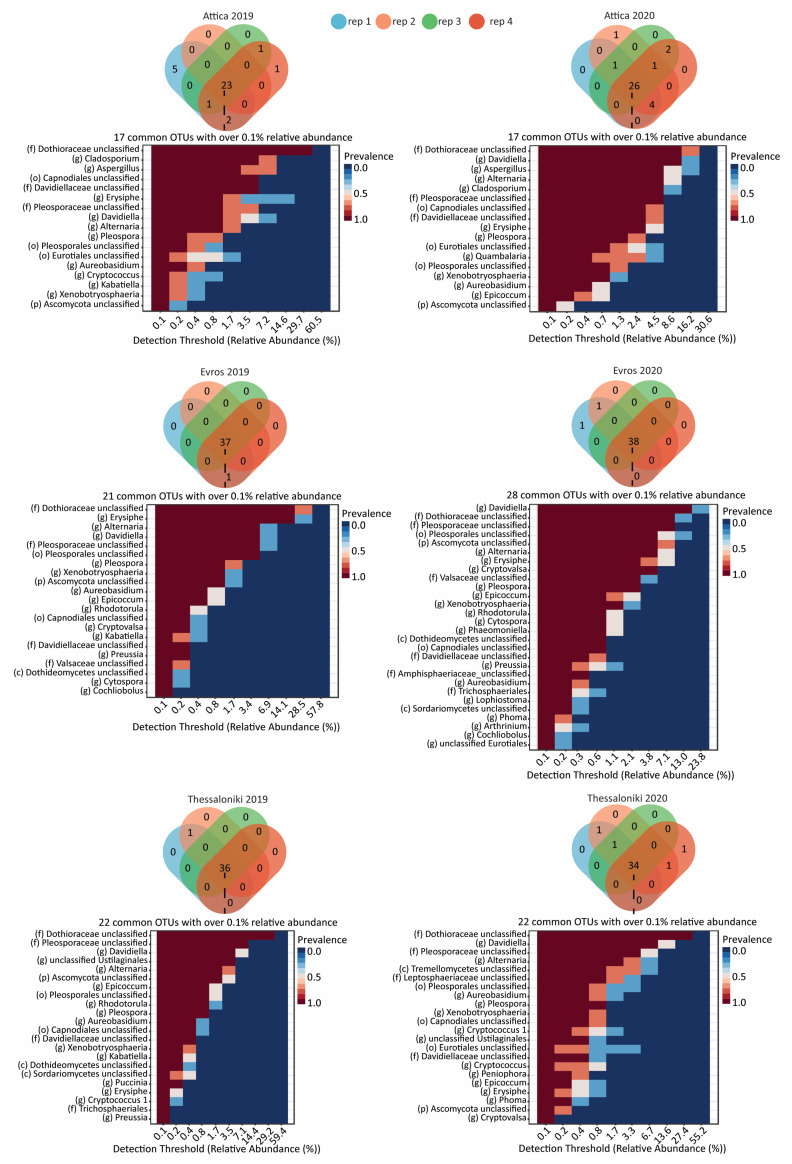
Venn diagrams depicting the shared fungal Operational Taxonomic Units (OTUs) within each vineyard and vintage, based on the analysis of four replicates. Additionally, the figure illustrates the relative abundance and prevalence of fungal OTUs exceeding 0.1% across the sampled datasets.

In addition, we identified the core fungal microbiome for each region and vintage (Figure 6). Unlike bacterial core microbiomes, the analysis of each dataset core microbiome showed that the region-specific fungal core microbiome of Assyrtiko encompasses, a significant proportion of the total identified OTUs (Figure 6). More specifically, in Attica, with a total of 33 and 35 OTUs identified for the two vintages, the core microbiome of 2019 comprised of 23 OTUs (69.9%) and that of 2020 of 26 OTUs (74.2%). For Evros samples, the core microbiomes for 2019 and 2020 consisted of 37 OTUs (97.3%) and 38 OTUs (95.0%), respectively. Similarly, for Thessaloniki, the fungal core microbiome comprised 36 OTUs (97.3%) for 2019 and 34 OTUs (89.4%) for 2020. Notably, more than 50% of the core microbiome OTUs were detected with an abundance higher than 0.1%; these findings suggest that the structure of fungal community across the samples collected from the same vineyard is characterized by considerable stability.

**Figure 7:**
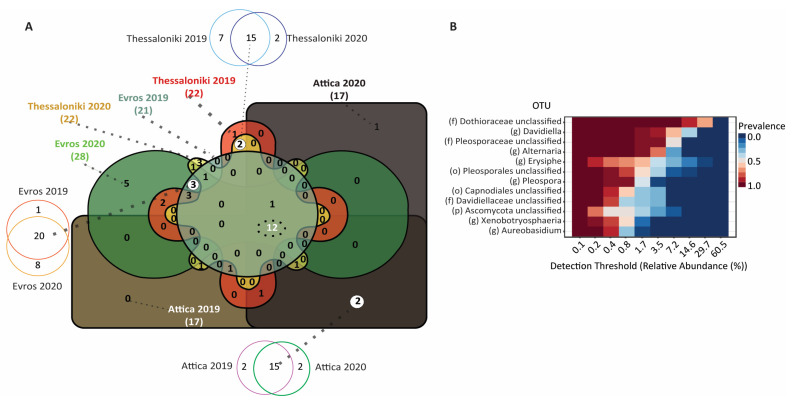
(**A**) Non-symmetric Venn diagram illustrating common and unique fungal OTUs within the different datasets. The core microbiome for each region in the two vintages, identifying the number of OTUs consistently present in the same vineyard/region over both years, is shown in the three symmetrical Venn diagrams (**top**, **bottom**, **left**). The number of OTUs unique for a region is shown in white circles in the non-symmetrical Venn diagram. (**B**) A diagram showing the relative abundance and prevalence of the core Assyrtiko fungal microbiome (12 OTUs identified on grapes of all regions and vintages).

We then investigated the region-specific core microbiome comprising of OTUs detected irrespective of the vintage (Figure 7). The analysis of each dataset’s core microbiome revealed that a significant percentage of OTUs detected contributes to a region-specific core microbiome. Specifically, 88.2% of the common OTUs in the samples AT19 and AT20 comprised the core microbiome of the Attica region (15 OTUs), 95.2% of EV19 and 71.4% of EV20 comprised the core microbiome of the Evros region (20 OTUs), and 68.1% of TH19 and 88.2% of TH20 comprised the core microbiome of the Thessaloniki region (15 OTUs) (Figure 7). To uncover a potential region-specific fungal signature, we identified within the fungal core microbiome unique OTUs found only in the specific region. More specifically, in Attica, 2 unique OTUs (*Cladosporium*, *Aspergillus*) were identified; in Evros, 3 were identified (*Cochliobolus*, *Valsaceae*, *Cytospora*); and in Thessaloniki, 2 were identified (*Cryptococcus* 1, unclassified *Ustilaginales*) (Figure 7). Finally, when we comparatively analyzed the core microbiomes across all regions and for both timepoints a set of 12 OTUs comprising the core fungal microbiome of Assyrtiko variety present on the surface of grapes irrespective of geographic region and vintage was identified (Figure 7).

## 4. Discussion

In recent years, wine makers have sought to develop wines with increased complexity and flavor diversity, providing a sense of regional authenticity and uniqueness, thereby adding high value to their products. In this vein, the microbial communities present on grape skin have attracted significant interest, as epiphytic microorganisms play vital roles influencing wine quality, especially in spontaneous fermentations [32,33]. A significant number of studies using mainly amplicon NGS, utilizing the bacterial small subunit ribosomal RNA gene (16S rRNA) and the fungal ITS1-5.8S rRNA-ITS2 to assess the structure of bacterial and fungal microbial communities, respectively, have demonstrated that this intricate ecosystem is influenced by several factors, including vine genotype, environmental conditions such as geography and climate, as well as, anthropogenic factors, particularly viticultural practices [3,13,25,34,35,36,37]. Additionally, in some instances, these analyses have pointed to correlations between specific metabolites that contribute to distinct wine characteristics and the presence of particular microbial taxa [23,36,37,38]. The analysis of grape microbiome may, therefore, be used either to identify regional “signatures” or to predict specific organoleptic or even functional characteristics [23,38,39,40,41]. In this context, the spatiotemporal analysis of the grape microbiomes provides useful insights, especially considering that most varieties, even indigenous ones, are typically grown across extended geographic areas.

Assyrtiko, a white grape variety that originated in the island of Santorini in the Aegean Sea, is one of the emblematic Greek varieties used in winemaking throughout Greece but also, to a lesser extent, in other countries such as Italy, Australia, and USA.

In this study, we characterized the spatiotemporal structure of the bacterial and fungal microbial communities of the Assyrtiko variety at two vintages and from three viticultural regions located across in Greece, namely Attica, Thessaloniki, and Evros, with distances between them ranging from approximately 240 to 360 km. Our results show that among the two factors that we studied, biogeography and vintage, biogeography has the strongest effect in both fungal and bacterial microbial communities.

The effect of biogeography that we observed was more evident in the fungal community. So far, a limited number of studies have focused on the comparative analysis of the epiphytic grape microbiome between viticulture regions with distances >150 km with results that are in accordance with our data [13,14,18,42]. However, the strong effect of biogeography on the structure of grape fungal communities at large geographical scale has been also observed in the microbiome analysis of must from viticulture regions in California, Australia, New Zealand, China, Cyprus, and Greece [3,17,25,43,44,45,46].

*Ascomycota* emerged as the predominant fungal phylum across all samples with *Basidiomycota* appearing in relatively smaller abundance. These results are in accordance with previous studies of grape microbiome [10,13,47] as well as of must microbiome [3,48]. Furthermore, the frequently observed in grape microbiome studies fungal genera such as *Davidiella*, *Alternaria*, *Erysiphe,* and *Aerobasidium* [10,47] are of the core fungal microbiome of Assyrtiko variety (Figure 7). Furthermore, despite *Aspergillus* being frequently encountered in the fungal microbial communities [10], in our study it was only detected in the samples from Attica. Notably, *Aspergillus* has been previously documented to exhibit such variability in its abundance on Chardonnay and Cabernet Sauvignon grapes, particularly in relation to the viticultural area [3].

While the bacterial community exhibited higher diversity compared to the fungal, the influence of biogeography on it at the large geographical scale, appeared to be weaker. Consistent with previous research [10,17,25], the prevailing phyla observed across all samples were *Proteobacteria* and *Actinobacteria*, with lower abundance of *Firmicutes*, *Bacteroidetes*, and *Acidobacteria*. Notably, we did not detect the phylum *Cyanobacteria*, which was found to be dominant among samples of the varieties Italian Riesling, Cabernet Franc, Pinot Blanc, and Riesling (LSL) [37]. Furthermore, the genus *Bacillus*, which has been described to stimulate plant growth and health [49], belongs to the core bacterial microbiome of Assyrtiko (Figure 4). This finding comes in accordance with other studies in which *Bacillus* was consistently detected in all samples using both high-throughput sequencing [37,50] and culture-dependent [49,51] methods. Furthermore, our analysis revealed a high abundance of the *Oxalobacteraceae* family, a member of the *Burkholderiales* order, in the Thessaloniki 2020. Intriguingly, the relative abundance of this family has been previously noted to vary between different vintage years [46] and viticulture regions for Cabernet Sauvignon, Merlot, Italic Riesling, and Cabernet Franc in China [13].

## 5. Conclusions

The spatiotemporal study of the grape microbial community of Assyrtiko variety across diverse regions and two vintages in Greece revealed both region and time specific patterns in terms of microbial diversity related to the *terroir*. Our study underscores the significant influence of biogeography on both fungal and bacterial microbial communities, with fungal communities more sensitive to biogeography. These findings align with existing research and contribute to a growing body of evidence highlighting the importance of considering regional differences in grape microbiomes and pave the way for the identification and further characterization of indigenous strains that can be exploited for the development of wines with distinct, regional organoleptic characteristics and high added value.

## Figures and Tables

**Table 1:** Alpha diversity and abundance estimation of the bacterial communities within the samples.

Region	Year	OTUs	Observed OTUs	Shannon	Simpson
Attica	2019	114	83.00 ± 8.04	3.40 ± 0.44	0.92 ± 0.06
Attica	2020	119	96.75 ± 6.24	3.53 ± 0.08	0.94 ± 0.01
Evros	2019	125	108.00 ± 1.41	3.63 ± 0.32	0.93 ± 0.04
Evros	2020	119	89.75 ± 12.31	3.53 ± 0.10	0.93 ± 0.01
Thessaloniki	2019	120	96.50 ± 11.09	3.58 ± 0.30	0.93 ± 0.03
Thessaloniki	2020	117	99.50 ± 2.89	2.81 ± 0.05	0.85 ± 0.02

**Table 2:** Alpha diversity and abundance estimation of the fungal communities within the samples.

Region	Year	OTUs	Observed OTUs	Shannon	Simpson
Attica	2019	33	26.75 ± 3.50	1.86 ± 0.20	0.73 ± 0.08
Attica	2020	35	31.75 ± 1.50	2.42 ± 0.09	0.87 ± 0.02
Evros	2019	38	37.50 ± 0.58	1.81 ± 0.22	0.71 ± 0.08
Evros	2020	40	38.75 ± 0.96	2.62 ± 0.07	0.89 ± 0.01
Thessaloniki	2019	37	36.50 ± 0.58	1.80 ± 0.08	0.67 ± 0.03
Thessaloniki	2020	38	36.00 ± 0.82	1.99 ± 0.11	0.73 ± 0.04

## Data Availability

The data presented in this study are available in Tables S2 and S3.

## Supplementary Materials

The following supporting information can be downloaded at: https://www.mdpi.com/article/10.3390/microorganisms12030577/s1, Supplementary Figure S1: Map of Greece showing the position of the vineyards; Supplementary Table S1: Geographic data and climatological information regarding the sites and dates of collection; Supplementary Table S2: Bacterial OTU tables; Supplementary Table S3: Fungal OTU tables.

## References

[1] Savo V., Kumbaric A., Caneva G. (2016). Grapevine (*Vitis Vinifera* L.) Symbolism in the Ancient Euro-Mediterranean Cultures. Econ. Bot..

[2] Alston J.M., Sambucci O. (2019). The Grape Genome.

[3] Bokulich N.A., Thorngate J.H., Richardson P.M., Mills D.A. (2014). Microbial Biogeography of Wine Grapes Is Conditioned by Cultivar, Vintage, and Climate. Proc. Natl. Acad. Sci. USA.

[4] Belda I., Ruiz J., Esteban-Fernández A., Navascués E., Marquina D., Santos A., Moreno-Arribas M.V. (2017). Microbial Contribution to Wine Aroma and Its Intended Use for Wine Quality Improvement. Molecules.

[5] Xu X., Miao Y., Wang H., Du J., Wang C., Shi X., Wang B. (2022). Analysis of Microbial Community Diversity on the Epidermis of Wine Grapes in Manasi’s Vineyard, Xinjiang. Foods.

[6] Wei R., Ding Y., Chen N., Wang L., Gao F., Zhang L., Song R., Liu Y., Li H., Wang H. (2022). Diversity and Dynamics of Microbial Communities during Spontaneous Fermentation of Cabernet Sauvignon (*Vitis Vinifera* L.) from Different Regions of China and Their Relationship with the Volatile Components in the Wine. Food Res. Int..

[7] Wei R.T., Chen N., Ding Y.T., Wang L., Liu Y.H., Gao F.F., Zhang L., Li H., Wang H. (2022). Correlations between Microbiota with Physicochemical Properties and Volatile Compounds during the Spontaneous Fermentation of Cabernet Sauvignon (*Vitis Vinifera* L.) Wine. LWT.

[8] Comitini F., Capece A., Ciani M., Romano P. (2017). New Insights on the Use of Wine Yeasts. Curr. Opin. Food Sci..

[9] Wei T.L., Zheng Y.P., Wang Z.H., Shang Y.X., Pei M.S., Liu H.N., Yu Y.H., Shi Q.F., Jiang D.M., Guo D.L. (2023). Comparative Microbiome Analysis Reveals the Variation in Microbial Communities between ‘Kyoho’ Grape and Its Bud Mutant Variety. PLoS ONE.

[10] Wei R.T., Chen N., Ding Y.T., Wang L., Gao F.F., Zhang L., Liu Y.H., Li H., Wang H. (2022). Diversity and Dynamics of Epidermal Microbes During Grape Development of Cabernet Sauvignon (*Vitis Vinifera* L.) in the Ecological Viticulture Model in Wuhai, China. Front. Microbiol..

[11] Barata A., Malfeito-Ferreira M., Loureiro V. (2012). The Microbial Ecology of Wine Grape Berries. Int. J. Food Microbiol..

[12] Nisiotou A.A., Rantsiou K., Iliopoulos V., Cocolin L., Nychas G.J.E. (2011). Bacterial Species Associated with Sound and Botrytis-Infected Grapes from a Greek Vineyard. Int. J. Food Microbiol..

[13] Gao F., Chen J., Xiao J., Cheng W., Zheng X., Wang B., Shi X. (2019). Microbial Community Composition on Grape Surface Controlled by Geographical Factors of Different Wine Regions in Xinjiang, China. Food Res. Int..

[14] Kioroglou D., Kraeva-Deloire E., Schmidtke L.M., Mas A., Portillo M.C. (2019). Geographical Origin Has a Greater Impact on Grape Berry Fungal Community than Grape Variety and Maturation State. Microorganisms.

[15] Martins G., Vallance J., Mercier A., Albertin W., Stamatopoulos P., Rey P., Lonvaud A., Masneuf-Pomarède I. (2014). Influence of the Farming System on the Epiphytic Yeasts and Yeast-like Fungi Colonizing Grape Berries during the Ripening Process. Int. J. Food Microbiol..

[16] Perazzolli M., Antonielli L., Storari M., Puopolo G., Pancher M., Giovannini O., Pindo M., Pertot I. (2014). Resilience of the Natural Phyllosphere Microbiota of the Grapevine to Chemical and Biological Pesticides. Appl. Environ. Microbiol..

[17] Pinto C., Pinho D., Sousa S., Pinheiro M., Egas C., Gomes A.C. (2014). Unravelling the Diversity of Grapevine Microbiome. PLoS ONE.

[18] Wei Y.J., Wu Y., Yan Y.Z., Zou W., Xue J., Ma W.R., Wang W., Tian G., Wang L.Y. (2018). High-Throughput Sequencing of Microbial Community Diversity in Soil, Grapes, Leaves, Grape Juice and Wine of Grapevine from China. PLoS ONE.

[19] Cocolin L., Heisey A., Mills D.A. (2001). Direct Identification of the Indigenous Yeasts in Commercial Wine Fermentations. Am. J. Enol. Vitic..

[20] Martins G., Miot-Sertier C., Lauga B., Claisse O., Lonvaud-Funel A., Soulas G., Masneuf-Pomarède I. (2012). Grape Berry Bacterial Microbiota: Impact of the Ripening Process and the Farming System. Int. J. Food Microbiol..

[21] Morgan H.H., du Toit M., Setati M.E. (2017). The Grapevine and Wine Microbiome: Insights from High-Throughput Amplicon Sequencing. Front. Microbiol..

[22] Belda I., Zarraonaindia I., Perisin M., Palacios A., Acedo A. (2017). From Vineyard Soil to Wine Fermentation: Microbiome Approximations to Explain the “Terroir” Concept. Front. Microbiol..

[23] Bokulich N.A., Collins T., Masarweh C., Allen G., Heymann H., Ebeler S.E., Mills D.A. (2016). Fermentation Behavior Suggest Microbial Contribution to Regional. mBio.

[24] Awad M., Giannopoulos G., Mylona P.V., Polidoros A.N. (2023). Comparative Analysis of Grapevine Epiphytic Microbiomes among Different Varieties, Tissues, and Developmental Stages in the Same Terroir. Appl. Sci..

[25] del Carmen Portillo M., Franquès J., Araque I., Reguant C., Bordons A. (2016). Bacterial Diversity of Grenache and Carignan Grape Surface from Different Vineyards at Priorat Wine Region (Catalonia, Spain). Int. J. Food Microbiol..

[26] Vlachos V.A. (2017). A Macroeconomic Estimation of Wine Production in Greece. Wine Econ. Policy.

[27] Nanou E., Mavridou E., Milienos F.S., Papadopoulos G., Tempère S., Kotseridis Y. (2020). Odor Characterization of White Wines Produced from Indigenous Greek Grape Varieties Using the Trained Assessors. Foods.

[28] Li H., Handsaker B., Wysoker A., Fennell T., Ruan J., Homer N., Marth G., Abecasis G., Durbin R. (2009). The Sequence Alignment/Map Format and SAMtools. Bioinformatics.

[29] Schloss P.D., Westcott S.L., Ryabin T., Hall J.R., Hartmann M., Hollister E.B., Lesniewski R.A., Oakley B.B., Parks D.H., Robinson C.J. (2009). Introducing Mothur: Open-Source, Platform-Independent, Community-Supported Software for Describing and Comparing Microbial Communities. Appl. Environ. Microbiol..

[30] Lu Y., Zhou G., Ewald J., Pang Z., Shiri T., Xia J. (2023). MicrobiomeAnalyst 2.0: Comprehensive Statistical, Functional and Integrative Analysis of Microbiome Data. Nucleic Acids Res..

[31] Love M.I., Huber W., Anders S. (2014). Moderated Estimation of Fold Change and Dispersion for RNA-Seq Data with DESeq2. Genome Biol..

[32] Cordero-Bueso G., Arroyo T., Serrano A., Tello J., Aporta I., Vélez M.D., Valero E. (2011). Influence of the Farming System and Vine Variety on Yeast Communities Associated with Grape Berries. Int. J. Food Microbiol..

[33] Griggs R.G., Steenwerth K.L., Mills D.A., Cantu D., Bokulich N.A. (2021). Sources and Assembly of Microbial Communities in Vineyards as a Functional Component of Winegrowing. Front. Microbiol..

[34] Canfora L., Vendramin E., Felici B., Tarricone L., Florio A., Benedetti A. (2018). Vineyard Microbiome Variations during Different Fertilisation Practices Revealed by 16s RRNA Gene Sequencing. Appl. Soil Ecol..

[35] Grangeteau C., Roullier-Gall C., Rousseaux S., Gougeon R.D., Schmitt-Kopplin P., Alexandre H., Guilloux-Benatier M. (2017). Wine Microbiology Is Driven by Vineyard and Winery Anthropogenic Factors. Microb. Biotechnol..

[36] Fernandes P., Afonso I.M., Pereira J., Rocha R., Rodrigues A.S. (2023). Epiphitic Microbiome of Alvarinho Wine Grapes from Different Geographic Regions in Portugal. Biology.

[37] Zhang J., Wang E.T., Singh R.P., Guo C., Shang Y., Chen J., Liu C. (2019). Grape Berry Surface Bacterial Microbiome: Impact from the Varieties and Clones in the Same Vineyard from Central China. J. Appl. Microbiol..

[38] Verginer M., Leitner E., Berg G. (2010). Production of Volatile Metabolites by Grape-Associated Microorganisms. J. Agric. Food Chem..

[39] Gilbert J.A., Van Der Lelie D., Zarraonaindia I. (2014). Microbial Terroir for Wine Grapes. Proc. Natl. Acad. Sci. USA.

[40] Mezzasalma V., Sandionigi A., Bruni I., Bruno A., Lovicu G., Casiraghi M., Labra M. (2017). Grape Microbiome as a Reliable and Persistent Signature of Field Origin and Environmental Conditions in Cannonau Wine Production. PLoS ONE.

[41] De Filippis F., La Storia A., Blaiotta G. (2017). Monitoring the Mycobiota during Greco Di Tufo and Aglianico Wine Fermentation by 18S RRNA Gene Sequencing. Food Microbiol..

[42] Papadopoulou E., Bekris F., Vasileiadis S., Krokida A., Rouvali T., Veskoukis A.S., Liadaki K., Kouretas D., Karpouzas D.G. (2023). Vineyard-Mediated Factors Are Still Operative in Spontaneous and Commercial Fermentations Shaping the Vinification Microbial Community and Affecting the Antioxidant and Anticancer Properties of Wines. Food Res. Int..

[43] Taylor M.W., Tsai P., Anfang N., Ross H.A., Goddard M.R. (2014). Pyrosequencing Reveals Regional Differences in Fruit-Associated Fungal Communities. Environ. Microbiol..

[44] Kamilari E., Mina M., Karallis C., Tsaltas D. (2021). Metataxonomic Analysis of Grape Microbiota during Wine Fermentation Reveals the Distinction of Cyprus Regional Terroirs. Front. Microbiol..

[45] Li R., Yang S., Lin M., Guo S., Han X., Ren M., Du L., Song Y., You Y., Zhan J. (2022). The Biogeography of Fungal Communities across Different Chinese Wine-Producing Regions Associated with Environmental Factors and Spontaneous Fermentation Performance. Front. Microbiol..

[46] Steenwerth K.L., Morelan I., Stahel R., Figueroa-Balderas R., Cantu D., Lee J., Runnebaum R.C., Poret-Peterson A.T. (2021). Fungal and Bacterial Communities of “Pinot Noir” Must: Effects of Vintage, Growing Region, Climate, and Basic Must Chemistry. PeerJ.

[47] Liu D., Howell K. (2020). Community Succession of the Grapevine Fungal Microbiome in the Annual Growth Cycle. Environ. Microbiol..

[48] Setati M.E., Jacobson D., Bauer F.F. (2015). Sequence-Based Analysis of the *Vitis Vinifera* L. Cv Cabernet Sauvignon Grape Must Mycobiome in Three South African Vineyards Employing Distinct Agronomic Systems. Front. Microbiol..

[49] Martins G., Soulas L., Soulas G., Masneuf-pomare I. (2013). Characterization of Epiphytic Bacterial Communities from Grapes, Leaves, Bark and Soil of Grapevine Plants Grown, and Their Relations. PLoS ONE.

[50] Leveau J.H.J., Tech J.J. (2011). Grapevine Microbiomics: Bacterial Diversity on Grape Leaves and Berries Revealed by High-Throughput Sequence Analysis of 16S RRNA Amplicons. Acta Hortic..

[51] Kačániová M., Kunová S., Felsöciová S., Ivanišová E., Kántor A., Puchalski C., Terentjeva M. (2019). Microbiota of Different Wine Grape Berries. J. Food Sci..

