# Supplementary figures and images for: Spatiotemporal Dynamics of Assyrtiko Grape Microbiota
Source: Microorganisms. 2024 Mar 14;12(3):577. doi: 10.3390/microorganisms12030577 (PMC10975888; doi:10.3390/microorganisms12030577)

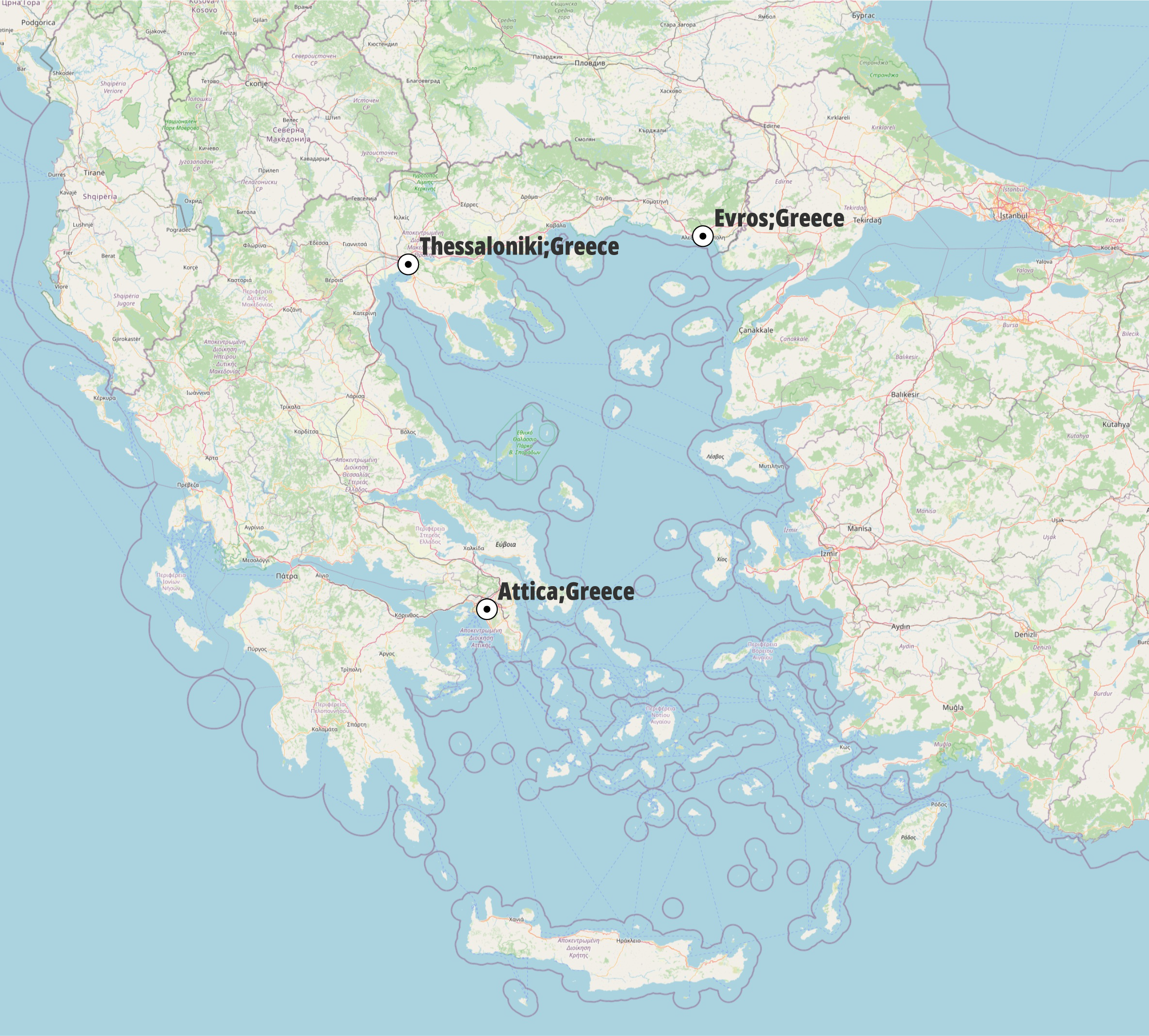

Supplement: Supplementary file 1 [file microorganisms-12-00577-s001.zip › Supplementary Figure S1.tiff]
